# Circle of Willis-Guided Localization for Simultaneous Detection and Classification of Large Vessel Occlusions in Brain CTA

**DOI:** 10.1007/s12021-026-09817-x

**Published:** 2026-09-16

**Authors:** Valeriia Abramova, Arnau Oliver, Uma M. Lal-Trehan Estrada, Rachika E. Hamadache, Paola Martínez Arias, Jordi Freixenet, Mikel Terceño, Yolanda Silva, Xavier Lladó

**Affiliations:** 1 Computer Vision and Robotics Institute, University of Girona, Girona, Catalonia Spain; 2 Department of Neurology, Hospital Universitari Dr Josep Trueta - Institut d’Investigació Biomèdica de Girona, Girona, Catalonia Spain

**Keywords:** Computed tomography angiography, Large vessel occlusion, Circle of willis, nnDetection

## Abstract

Large vessel occlusions (LVOs) are blockages in the brain’s major arteries that can cause severe neurological damage. Rapid and accurate detection using computed tomography angiography (CTA) is critical for timely stroke treatment. Here, we present a fully automated approach that detects LVOs and classifies the affected vessel simultaneously. Our method incorporates a spatial prior by using Circle of Willis (CoW) segmentation as additional input, guiding the model to anatomically relevant regions. We evaluated the two strategies, the global approach using the full CTA volume, and the local one focused on CoW regions. Both achieved high performance. Detection sensitivity was 0.97 at 0.20 false positives per image for the global approach, and 0.97 at 0.13 false positives for the local approach. Classification accuracy reached 94% and 91% for global and local strategies, respectively. Importantly, the local approach was 3.3*× * faster, offering a computationally efficient solution, a critical advantage in acute stroke care, where every minute impacts patient outcomes.

## Introduction

Stroke is the second most common cause of death and the third most common cause of death and disability combined (Fan et al., 1990). Ischemic stroke, which is caused by reduced or blocked blood supply to the part of the brain, accounts for *70%* of all stroke cases and it has a high risk of being repeated again (Clerigues et al., 2020; Fan et al., 1990). Such reduction of blood flow can be caused by the blockage of major vessels of the brain, namely Large Vessel Occlusion (LVO). *24%* to *46%* of acute ischemic strokes can happen because of LVO, and, as these occlusions are located in proximal vasculature, significant brain regions can be damaged, which results in a high mortality and morbidity, since less than *50%* of patients achieve an independent functional status at 3 months, despite endovascular treatments (Goyal et al., 2016; Nicholls et al., 2022). Therefore, it is important to act fast in the diagnosis and treatment of LVO-related strokes to reduce the effect of possible brain damage (Chen et al., 2023; Feng et al., 2021; Sen et al., 2024).

Brain imaging with computed tomography angiography (CTA) is the current gold standard for LVO diagnosis (Nicholls et al., 2022). However, interpreting CTA scans can be time-consuming and challenging, as radiologists must rapidly review large three-dimensional image volumes, often inspecting them slice-by-slice in multiple planes to carefully assess the intracranial vasculature for subtle signs of arterial occlusion, and account for normal anatomical variations and potential imaging artifacts. These challenges must be addressed under considerable time pressure, as every minute of delay in diagnosis and treatment is associated with poorer neurological outcomes (Powers et al., 2019). Therefore, automated analysis tools can highly assist clinicians to make faster decisions about the suspected stroke patients (Amukotuwa et al., 2019). Most existing research on LVO detection treats the problem as binary, focusing solely on whether an LVO is present (Kuang et al., 2025; Meng et al., 2022; Stib et al., 2020), as in the Image Analysis for CTA Endovascular Stroke Therapy Data Challenge (IACTA-EST^1^). In these works, detection identifies presence or absence of LVO without specifying its exact location. In contrast, our approach aims to precisely localize the LVO and identify the specific vessel segment involved. Even though all types of LVO are nowadays typically treated with endovascular treatment, the strategy can differ based on the occluded vessel segment because of different disease pathogenesis. For example, for occlusions in posterior circulation (e.g. basilar artery) balloon angioplasty and permanent stents are more frequently used, while in anterior circulation it is more common to perform stent thrombectomy and catheter aspiration (Sun et al., 2022). Additionally, there is limited data on optimal anesthesia type and endovascular treatment strategy for occlusions in posterior circulation (Heit et al., 2024; Terceño et al., 2023) and new clinical recommendations appear (Heit et al., 2024; Shirakawa et al., 2025). Therefore, automatic identification of vessel segment affected by occlusion is crucial in clinical practice.

Different commercial software solutions for LVO identification are currently available, including RapidLVO (iSchemaView, USA), Viz-LVO (Viz.ai, USA), e-CTA (Brainomix, UK), and StrokeViewer-LVO (Nicolab, Netherlands); however, despite their clinical utility, these systems remain limited by reduced sensitivity for distal occlusions, susceptibility to image artifacts leading to false positives, and reliance on standardized CTA acquisition protocols, which may hinder generalizability across institutions. As a result, research in this area remains highly active, with several studies moving beyond binary detection toward explicit LVO localization. Bathla et al. (2022) proposed a two-stage CNN-based pipeline for LVO detection on 4D-CTA, combining a classification network with a U-Net-based detector guided by a manually defined region of interest around the skull base and the expected trajectory of the Circle of Willis (CoW). Bagcilar et al. (2023) and Brugnara et al. (2023) employed the nnDetection framework (Baumgartner et al., 2021) to localize vessel occlusions in CTA, using maximum intensity projection images and bounding boxes spanning the affected vessel segments, with the latter demonstrating superior performance compared to commercial solutions. Other approaches have focused on specific vascular territories, such as the deep learning model by Kumar et al. (2023) targeting middle cerebral and internal carotid artery occlusions, while more recently Russo et al. (2026) combined GravityNet with a multi-head self-attention mechanism for LVO detection.

It should be noted that while several methods provide spatial localization of the occlusion in the form of bounding boxes, they typically do not associate the detected occlusion with a specific vessel segment, but instead provide only generic spatial localization of the affected region. This distinction is important, as identifying the vessel segment affected by LVO will improve anatomical interpretability, support more precise clinical assessment, guide intervention planning, and refine prognosis estimation.

In this work, we propose an approach that simultaneously detects LVOs and classifies the affected vessel segment, namely the basilar artery, terminal internal carotid artery, and middle cerebral artery. We further investigate the impact of using either the full CTA volume or a restricted region corresponding to the Circle of Willis (CoW) as model input. The main contributions of this work are as follows. First, we adapt the state-of-the-art self-configuring nnDetection framework to jointly perform LVO detection and vessel-specific classification, producing both a bounding box for the occlusion and a class label for the affected artery. To the best of our knowledge, this is the first study to address these two tasks within a single unified framework. Second, we incorporate an automated CoW segmentation to define an anatomically informed region of interest, thereby constraining the training space. We show that leveraging this spatial prior achieves performance comparable to full-volume training while substantially reducing processing time, a critical advantage in acute stroke care, where rapid and informed decision-making is essential.

## Methods

### Dataset

The development dataset comprised 179 CT angiography images of varying sizes and voxel spacings, all containing LVOs and acquired at Hospital Dr. Josep Trueta in Girona, Spain. All examinations were performed using a Philips Healthcare Ingenuity CT scanner. The dataset included cases with occlusions in the basilar artery, terminal internal carotid artery (TICA), and middle cerebral artery (M1 segment). LVO instances were annotated with 3D bounding boxes encompassing the thrombus by an expert neurologist from the collaborating hospital, based on the inspection of multiple CTA views.

**Fig. 1:**
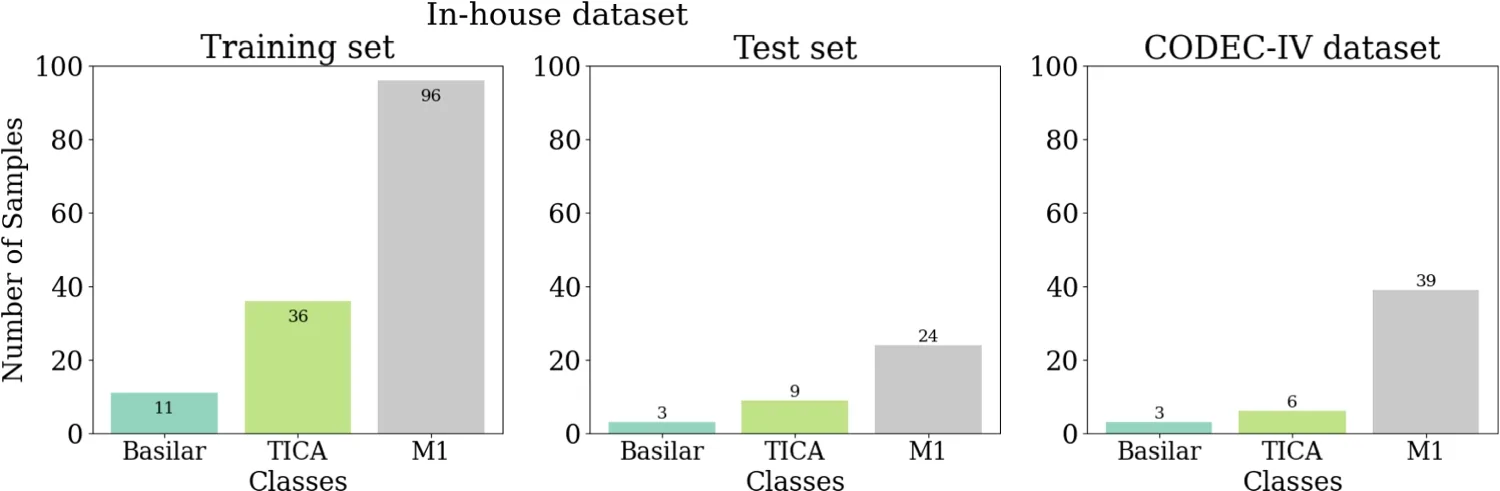
Distribution of classes in the development set and independent test set

The images were divided into two subsets. A total of 143 images were used for training, with the following class distribution: basilar artery (8% of cases), TICA (25%), and M1 (67%). The remaining 36 images were reserved for testing and were selected to preserve the same class distribution as in the training set. The class distributions for both subsets of the development dataset are shown in Fig. 1.

As an independent test set, we used the recently proposed CODEC-IV dataset (Werdiger et al., 2023). This dataset consists of 159 CTA images derived from CT perfusion (CTP) images, acquired on a Toshiba Aquilion scanner from several hospital sites. For the fair comparison, we chose 48 cases from this dataset which included LVOs in the same vessel segments as our in-house development dataset. Initially, the clot annotation was provided as an ellipse, which we extended to a bounding box to be compatible with our training pipeline. 

From Fig. 1 we see that both our in-house dataset and the independent test set are highly imbalanced with a great prevalence of M1 cases and only a few examples of basilar LVOs. This follows the natural distribution of common LVO locations and it is shown that M1 is usually the most frequent occluded vessel, while the occlusions in posterior circulation (including basilar artery) are much less common than in anterior circulation (Roland et al., 2025; Waqas et al., 2020).

A key difference between both datasets is the ground truth annotation. While the bounding box annotations of the development dataset were targeting at capturing the whole extent of the clot, the annotations of the independent test set were rather focusing on clot localization, indicating as accurately as possible the site of the occlusion. Therefore, the size of the ground truth bounding boxes of the CODEC-IV dataset was fixed for all the cases, whereas the sizes of the ground truth bounding boxes of the in-house dataset varied. The comparison of ground truth annotations for both datasets is shown in Table 1.

**Table 1:** Distribution of ground truth bounding boxes sizes for in-house development dataset and independent test set (CODEC-IV). The size of bounding boxes of CODEC-IV is fixed throughout the whole dataset

		Box height	Box width	Box depth	Box volume
Dataset	No. of images	(voxels)	(voxels)	(voxels)	(voxels$^3$)
		7 – 44	3 – 60	6 – 69	240 – 6336
In-house	179	(*17± 7*)	(*12± 7*)	(*16± 9*)	(*4751 ± 8596*)
CODEC-IV	48	10	10	6	600

All the images from both datasets were preprocessed prior to our study. The images were automatically cropped to include only the head of the patient and were skull-stripped using FSL toolbox (Jenkinson et al., 2002; Jenkinson and Smith, 2001). Moreover, we clipped the intensity values of the brain between 0-200 HU.

### Identification of the Circle of Willis

The Circle of Willis is a critical network of arteries located at the center of the brain (Sun et al., 2024; Yang et al., 2026). Given that it includes vessels that are affected by occlusions in our dataset, we utilized the spatial information provided by the CoW’s location to crop the input CT angiography images, thereby focusing the area for LVO detection.

However, segmentation of the CoW is a labor-intensive process, and ground truth vessel masks were unavailable for our datasets. To solve this issue, we used the CT angiography data of the “Topology-Aware Anatomical Segmentation of the Circle of Willis for CTA and MRA” challenge (TopCoW), held in 2023 and 2024 (Yang et al., 2026), which encompasses diverse anatomical CoW variants, to train a standard nnU-Net model (Isensee et al., 2020) using a 5-fold cross-validation approach. With this model, we achieved a Dice score of *0.944± 0.026* in the CoW CTA segmentation task.

**Fig. 2:**
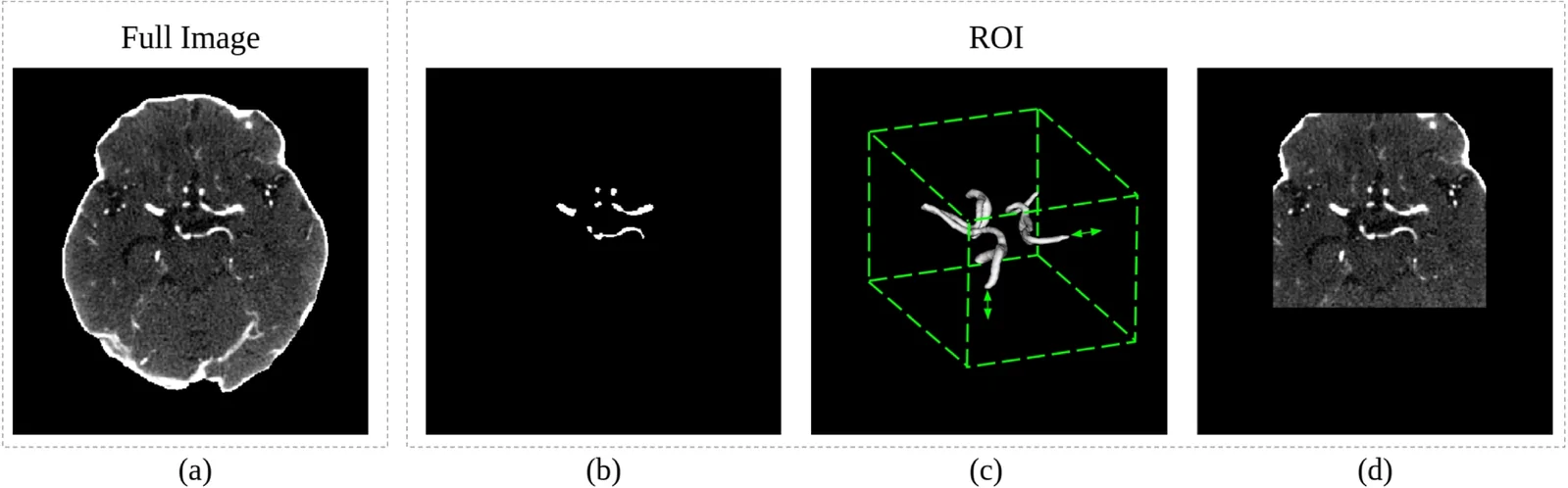
Illustration of the input images used. (**a**) Experiment using the full-sized CTA images as input for the model; (**b**) automatic segmentation of the Circle of Willis; (**c**) 3D region of interest (ROI) that we used to crop the original images, creating an (**d**) input for the second experiment

Subsequently, we applied the model weights to segment the Circle of Willis in the images from our datasets (Fig. 2b). To ensure that the entire CoW was fully captured and consider possible segmentation errors, we expanded the resulting automatic segmentation by 50 voxels in all three dimensions. This margin was determined based on the training data, ensuring that all occlusions present in our dataset were included in the region of interest (ROI, Fig. 2c). This process resulted in a binary mask that was used to crop the ROI for all images in both the training dataset and the independent test set (Fig. 2d).

### Network Architecture

**Fig. 3:**
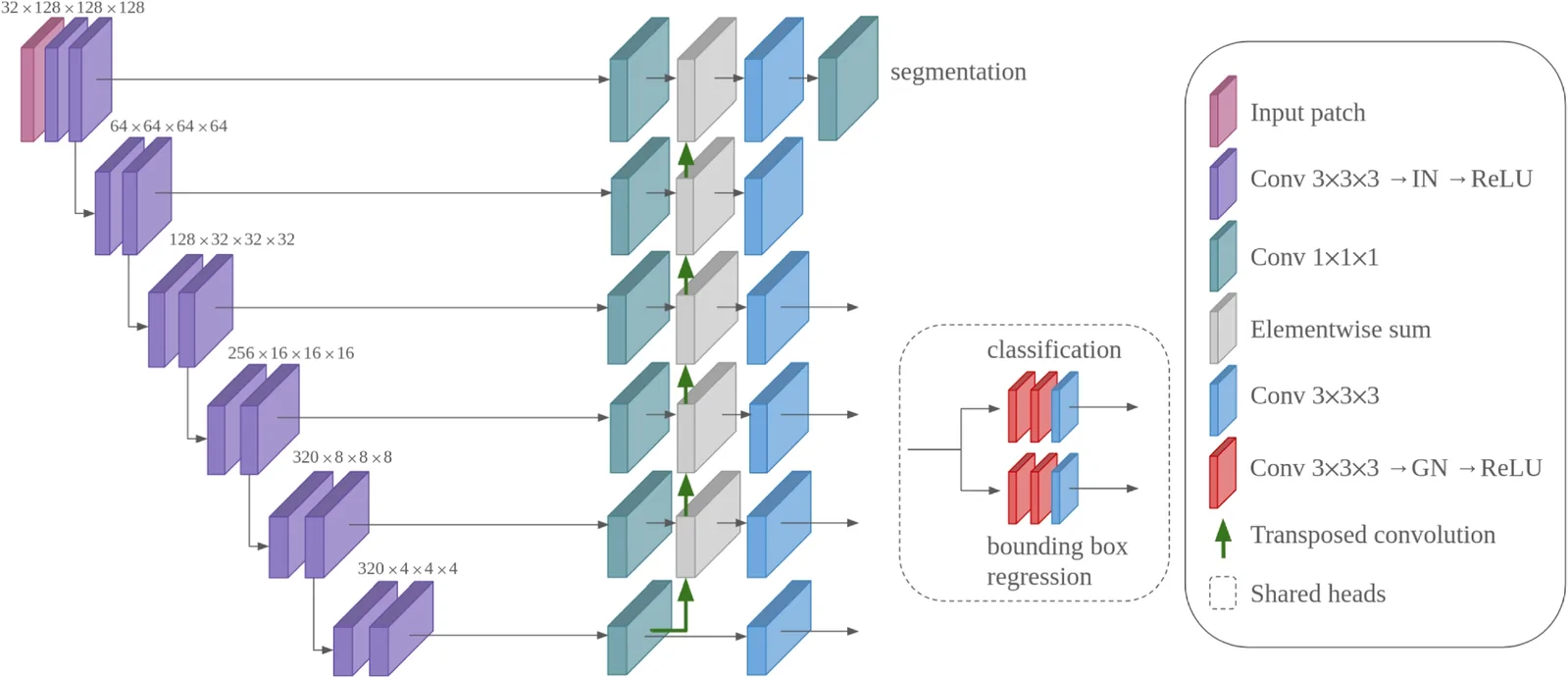
The schematic illustration of Retina U-Net architecture, which serves as a backbone to nnDetection framework. It combines the Retina Net one-stage detector with the U-Net architecture. Encoder is used to extract multi-level image features. Feature pyramid network (FPN) combines features from encoder and upsampled feature maps. Shared detection head is used for bounding box regression and classification; and a segmentation head is used to perform additional segmentation supervision

In our study we used the state-of-the-art 3D self-configuring nnDetection method (Baumgartner et al., 2021). This is based on the Retina U-Net architecture, proposed by Jaeger et al. (2018), which is a combination of the RetinaNet one-stage detector with a U-Net architecture used for semantic segmentation (Fig. 3). In this resulting network, each layer of the encoder consists of two $3\times 3\times 3$ convolutional layers each followed by instance normalization and ReLU activation function. The lateral connections are composed of single $1\times 1\times 1$ convolutional layers, while each decoder level consists of transposed convolutional layers. There are two sub-networks operating on certain pyramid levels of the Retina U-Net for the classification and for the bounding box regression. Each sub-network comprises two blocks of $3\times 3\times 3$ convolutional layers, followed by group normalization and ReLU activation. The classification head also includes a sigmoid function. The classification branch was trained with binary cross-entropy loss, while the regression branch was trained with generalized intersection-over union loss. Moreover, semantic segmentation supervision was incorporated during the training process.

The hyperparameters of the model were based on a standard 3D nnDetection configuration: (*a*) the number of epochs was set to 60 with batch size 4; (*b*) the initial learning rate was linearly increased to 0.01 over the first 4000 iterations and then followed a polynomial decay schedule; (*c*) we used a stochastic gradient descent optimizer with Nesterov momentum of 0.9. Instead of using automatically configured patch size, provided by nnDetection, we fixed it to $128\times 128\times 128$ to be consistent across the experiments and to be able both to capture global context information and to fit into the cropped images.

#### Implementation Details

Our approach was implemented using PyTorch 1.13.1 and experiments were run on an NVIDIA GeForce GTX 1080 Ti GPU with 12 GB RAM.

## Results

### Experimental Setup

**Fig. 4:**
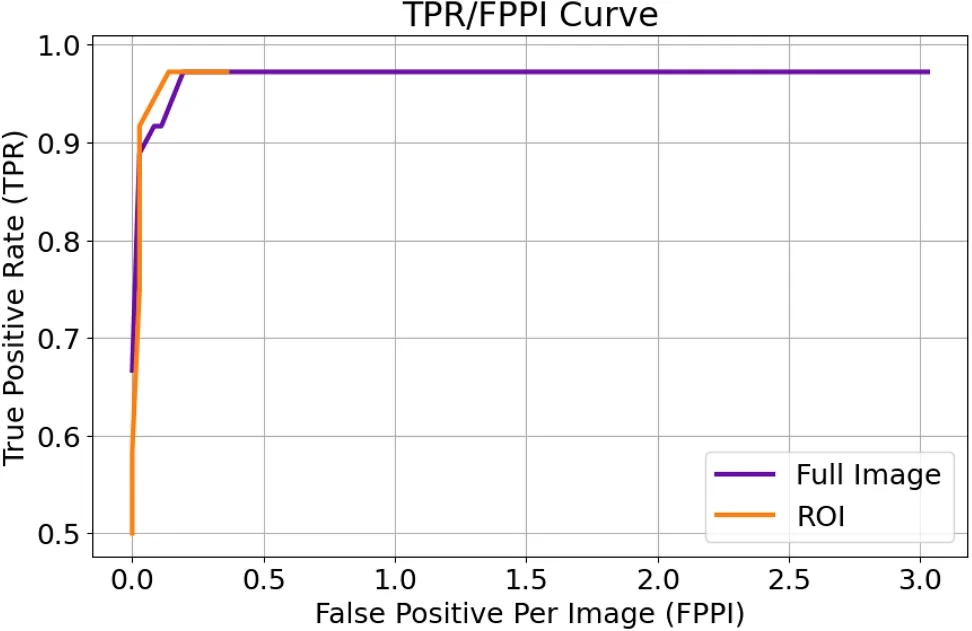
FROC analysis for LVO detection sensitivity for the in-house test set (36 cases). Here, the purple curve shows results of inference using an ensemble of 5 models trained on full-sized images, while the orange curve shows results of inference using an ensemble of 5 models trained on input images cropped around Circle of Willis region

Detection performance was evaluated using a free-response receiver operating characteristic (FROC) analysis. A predicted bounding box was considered a true positive (TP) if it achieved at least a *10%* intersection over union (IoU) with the corresponding ground truth, in line with previous studies (Brugnara et al., 2023). Predictions with a confidence score below 0.1 were discarded. When multiple TP predictions were present for a single case, only the TP with the highest confidence score was retained, and the corresponding bounding box was used to assign the final class label. To compare detection performance across methods, we evaluated the binary detection outcomes for each individual case at a fixed operating point of 0.1 FPPI. A case-level paired bootstrap analysis (2000 resamples) was used to estimate the 95% confidence interval (CI) for the difference in sensitivity between the two approaches. Classification performance was assessed by computing confusion matrices for all vessel classes included in the dataset. Agreement between classifiers was quantified using Cohen’s kappa (*κ *) statistic (Cohen, 1960).

We evaluated the performance of our approach under two scenarios: an unseen internal dataset (36 images) and an external dataset (48 images), the latter used to assess generalization. The approach was trained using a 5-fold cross-validation scheme on the training dataset (143 images), with 80% of the images (four folds) used for training and the remaining 20% (one fold) for validation in each iteration. Inference on both test datasets was performed using an ensemble of the five models, with the weighted average serving as the final decision criterion.

For each case, two experiments were conducted to analyze the impact of restricting the training to the Circle of Willis (CoW) region. In the baseline experiment, the network was trained using the original full-sized input images, referred to as the global approach. In the second experiment, images were cropped around the CoW-based region of interest (ROI) and used as input to the model, following the same cross-validation split, referred to as the local approach.

### First Scenario: Internal Dataset

The FROC curves for the detection task are shown in Fig. 4. The curves were obtained for held-out test set derived from our in-house dataset for global and local (ROI-cropped) approaches trained in a 5-fold cross-validation manner. The global approach achieved a sensitivity of 0.92 at 0.08 false positives per image, and a sensitivity of 0.97 at 0.2 false positives per image. Meanwhile, the cropped ROI approach achieved a sensitivity of 0.92 at 0.03 false positives per image, and a sensitivity of 0.97 at 0.13 false positives per image.

From these findings, we conclude that the network performed similarly when constrained to the region of interest compared to using the full image context. At 0.1 FPPI, the case-level paired bootstrap yielded a mean sensitivity difference (local - global) of 0.000 with a *95%* CI of [-0.081, 0.081], indicating no systematic difference between the two approaches. Interestingly, the model trained on full images generated more false positive predictions with lower confidence scores, whereas the model trained on the ROI produced fewer false positives. Moreover, both approaches missed only a single occlusion while detecting the rest correctly.

To complement the overlap-based evaluation, we additionally assessed localization accuracy by computing the 3D Euclidean distance between the centers of the predicted and ground truth bounding boxes. On the internal test set, the mean center-to-center distance was 1.76 ± 1.49 mm (3.07 ± 2.33 voxels) for the global approach and 1.76 ± 1.32 mm (3.07 ± 2.09 voxels) for the local approach, indicating that both approaches accurately localize the occlusion site.

**Fig. 5:**
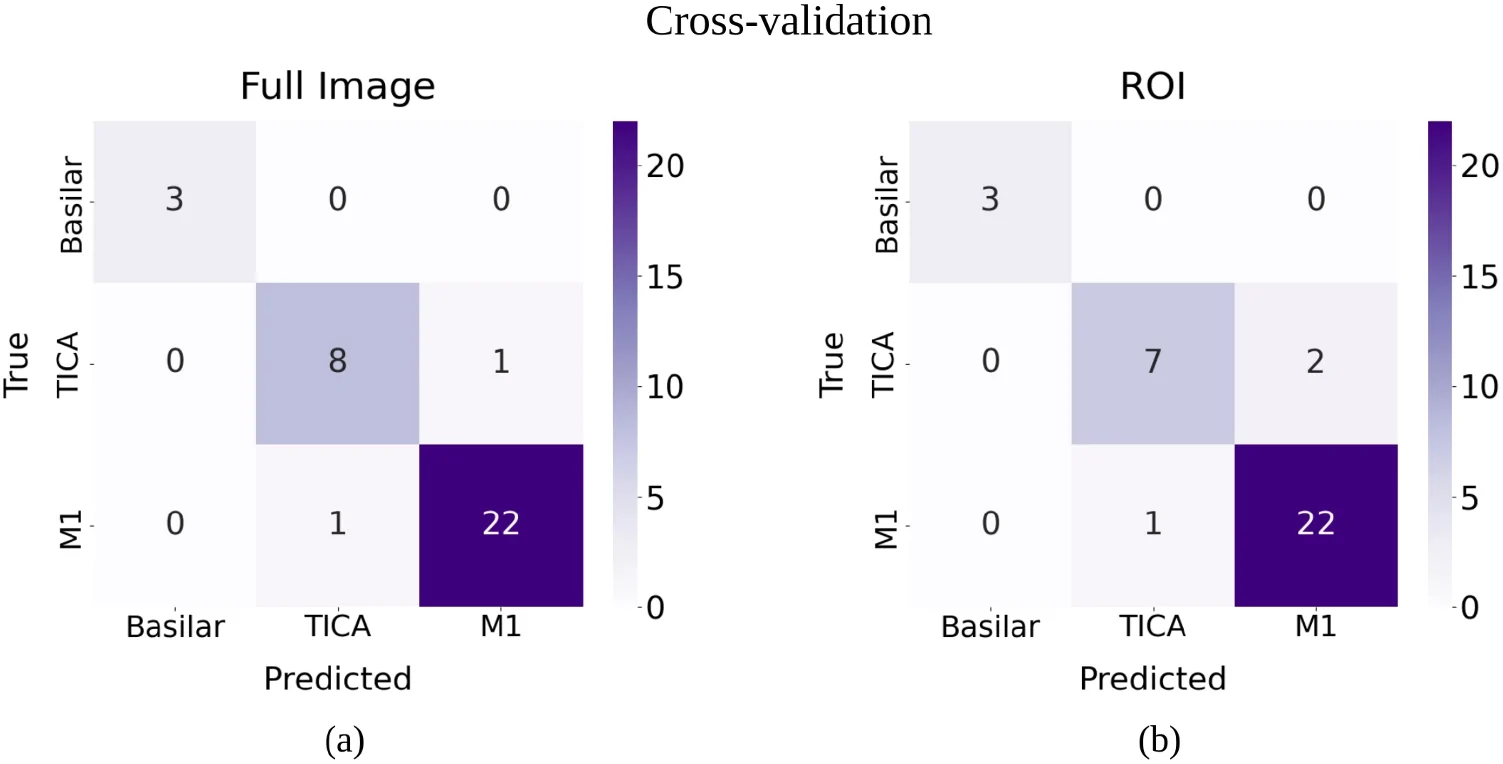
Confusion matrices displaying the classes predicted by nnDetection versus the ground truth labels in the in-house test dataset. (**a**) shows the experiment where the training was performed on full-sized images (*κ =0.88*); (**b**) shows an experiment where the training was performed on the region of interest (*κ =0.82*)

The classification results of the algorithm are shown with confusion matrices in Fig. 5. Regardless of whether the models were trained globally or locally, most classes were correctly predicted. Overall, the class-wise accuracy is almost the same for both approaches, except for TICA where accuracy was *89%* when trained with the full image and *78%* when training with the ROI, obtaining almost perfect agreement with *κ =0.88* and *κ =0.82* with regard to the ground truth, respectively. Additionally, in both the global and local approaches, all LVOs in the basilar artery were classified correctly. This is likely due to the basilar artery’s distinctive location at the base of the brain, centrally positioned and separated from other vessels, which reduces ambiguity in classification. Comparing the agreement between both models we obtained *κ =0.94*, demonstrating an almost perfect agreement between both approaches.

When accounting for detection failures, the overall proportion of all cases in which the occlusion was both detected and correctly classified was 33/36 for the global approach and 32/36 for the local approach. All basilar artery cases were correctly detected and classified by both approaches. For TICA, the proportion was 8/9 for the global approach and 7/9 for the local approach, while for M1 both approaches achieved 22/24.

### Second Scenario: Independent Dataset

**Fig. 6:**
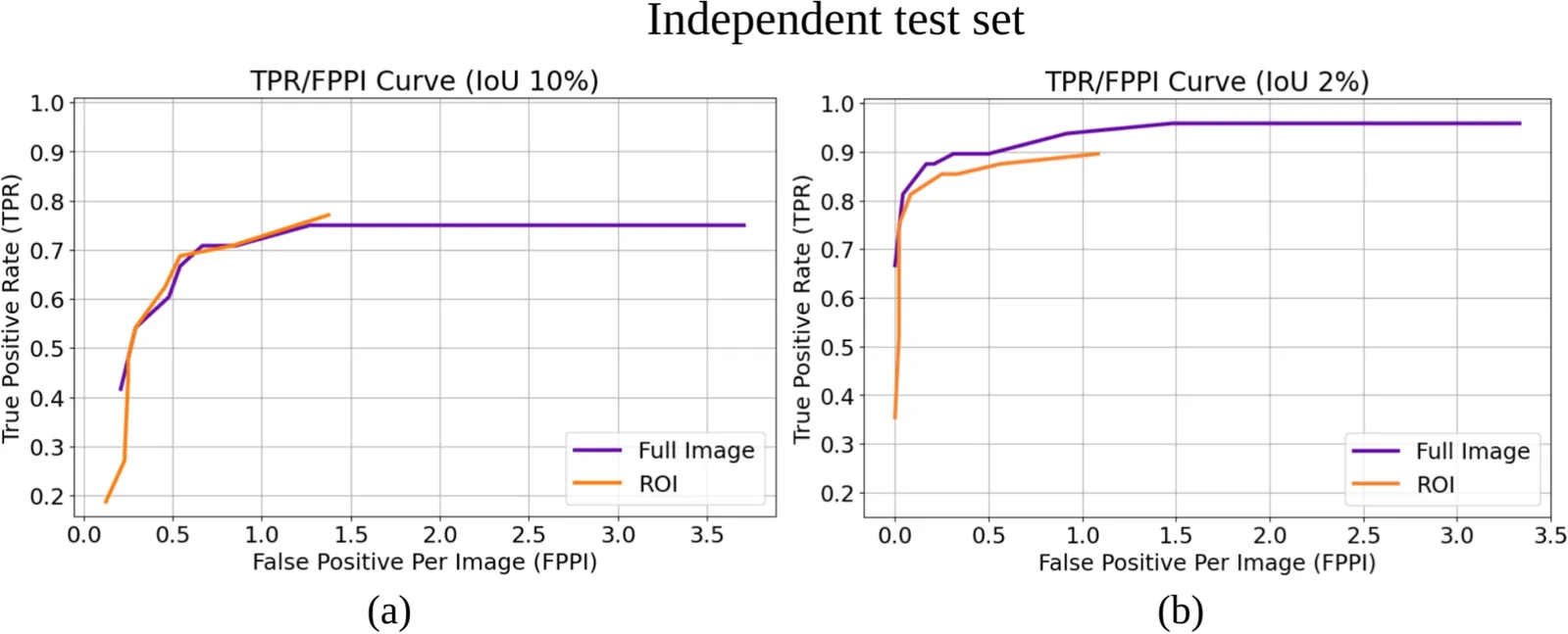
FROC analysis for LVO detection sensitivity for independent test set (48 cases). (**a**) shows FROC analysis with the initially defined criterion for true positive bounding box to have the *10%* IoU with the ground truth box. (**b**) shows the FROC analysis in case when we lower the threshold for the true positive bounding box to *2%* IoU with ground truth bounding box. On both subfigures, purple curves show results of inference using an ensemble of 5 models trained on full-sized images. The orange curves show the results of inference using an ensemble of 5 models trained on the images cropped around Circle of Willis region

To evaluate the performance of our model on the independent test set, we used again the weighted average ensemble of the 5 models generated from the cross-validation strategy for both global and local experiments. The resulting FROC curves for the detection task are shown in Fig. 6(a). Our approach achieved a sensitivity of 0.71 at 0.67 false positives per image for the global approach and the same sensitivity at 0.83 false positives per image for the local approach. At 0.1 FPPI, the case-level paired bootstrap yielded a mean sensitivity difference of -0.022 with a 95% CI of [-0.159, 0.114], indicating no systematic difference between the two approaches.

Comparing the results with those obtained on the internal test set, we observed a drop in detection performance on the CODEC-IV dataset. One likely reason for this decrease is the substantial mismatch between the sizes of the ground truth bounding boxes in the development and independent test datasets. In anchor-based object detection models, the selection of predefined anchors, which represent potential bounding box positions, is crucial, as it affects both training and inference by injecting prior knowledge about object sizes into the model. Our nnDetection framework selects initial anchors based on the bounding box sizes in the training dataset. As shown in Table 1, bounding boxes in the training set not only vary significantly but are mostly larger than the ground truth boxes in the CODEC-IV dataset. Consequently, even when predicted bounding boxes are correctly positioned over the clot, their larger size can reduce the intersection over union (IoU), which may prevent them from being considered true positives.

To account for this, we also evaluated detection performance on the independent test set using an IoU threshold of 2% for true positives. The resulting FROC curves are shown in Fig. 6(b). In this setting, the global approach achieved a sensitivity of 0.90 at 0.50 false positives per image, while the local approach achieved 0.88 sensitivity at 0.56 false positives per image. These results indicate that our model accurately predicted the LVO locations on the independent test set, with bounding box sizes consistent with those in the development dataset.

We further assessed localization accuracy by computing the 3D Euclidean distance between the centers of the predicted and ground truth bounding boxes. On the independent test set, the mean distance was 2.47 ± 1.13 mm (5.38 ± 2.37 voxels) for the global approach and 2.43 ± 1.23 mm (5.39 ± 2.74 voxels) for the local approach, showing that both models consistently localize the center of the occlusion.

Comparing the performance on the CODEC-IV dataset with the results obtained on the internal test set, both ensembles maintained high sensitivity on the independent test set with a slight increase in the false positive rate. Moreover, as expected, inference on ROI-cropped test cases produced less false positives compared to inference on full-sized images.

**Fig. 7:**
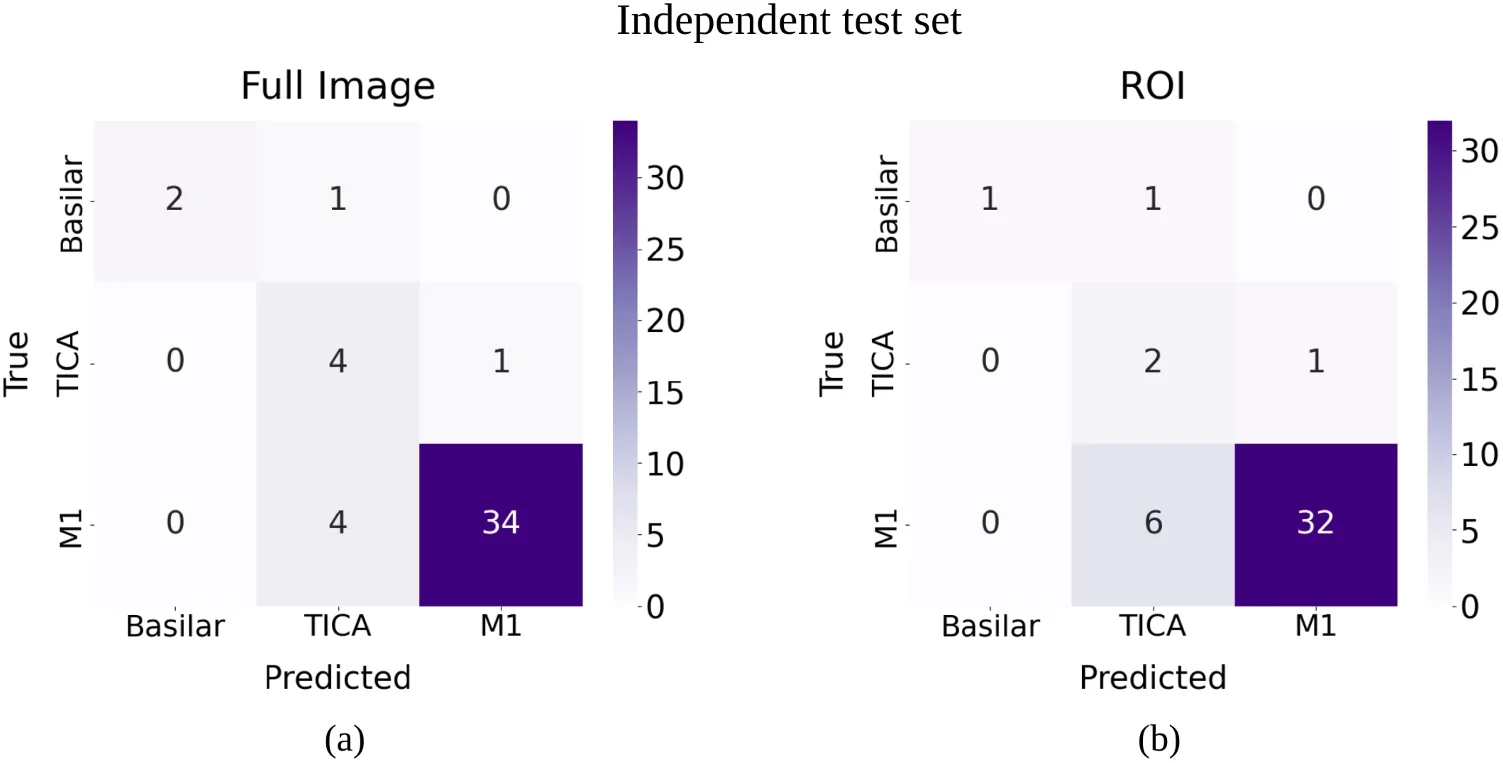
Confusion matrices displaying the classes predicted by nnDetection versus the ground truth labels in an independent test set. (**a**) shows the experiment where the training was performed on full-sized images and inference done on the independent test set (*κ =0.62*); (**b**) shows an experiment where the training was performed on the region of interest and inference done on the independent test set (*κ =0.40*)

**Fig. 8:**
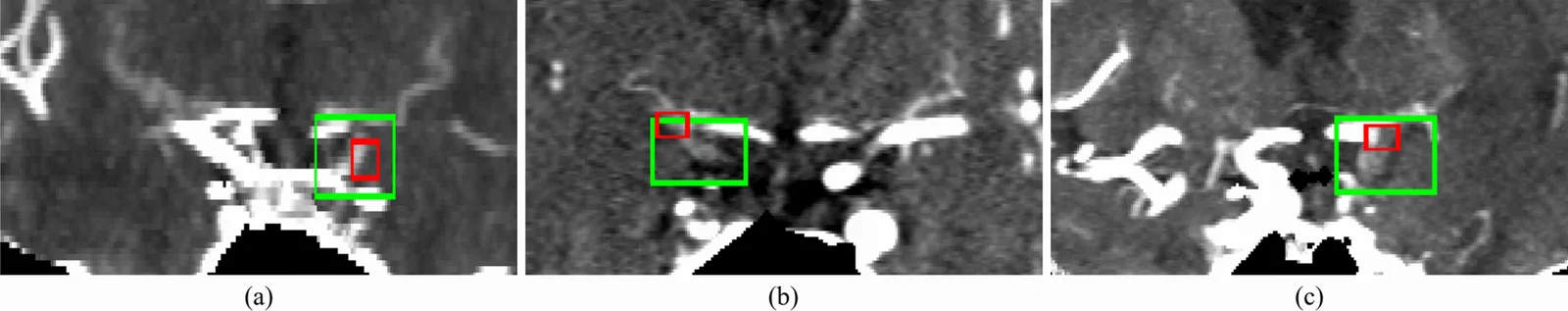
Examples of resulting bounding boxes obtained in our experiments for the independent test set. The colors of the borders represent ground truth bounding box (red) and predicted bounding box (green). (**a**) and (**c**) are maximum intensity projection images. In all (**a**), (**b**) and (**c**) LVOs are labeled as M1, however our models (both global and local) misclassified them as TICA since the predicted bounding boxes captured the whole extent of the clot from TICA onward

The classification results obtained for the independent test set are shown in Fig. 7. We observed a similar behavior than that of the internal test dataset, classifying correctly most of the cases. Misclassifications happened between neighboring classes (e.g. TICA vs M1). Some visual examples are shown in Fig. 8. Analyzing these wrongly classified bounding boxes we noticed that our models indeed were capturing the whole extent of the clot, not only the occlusion site, which could lead to ambiguous classification results. As shown in the examples, all the ground truth bounding boxes were assigned to M1 vessel segment, and their placement supports the choice of such classification label. However, both our local and global models captured the whole extent of the clots originating in terminal carotid artery prior to reaching M1 segment and therefore these boxes were classified as TICA-located occlusions. Nevertheless, it is important to remark that despite complicated and vague cases with occlusions near branching points, both global and local models showed robust classification performance of the predicted bounding boxes in the independent test set.

When accounting for detection failures, the overall proportion of all cases in which the occlusion was both detected and correctly classified was 40/48 for the global approach and 35/48 for the local approach. For M1, the proportion was 34/39 for the global approach and 32/39 for the local approach. For TICA, the proportion was 4/6 for the global approach and 2/6 for the local approach, while for the basilar artery it was 2/3 and 1/3, respectively. We note that only 3 and 6 cases were available for basilar and TICA, respectively, and these results should be interpreted with caution given the very small sample sizes.

## Discussion

In this study, we adapted the well-known nnDetection framework to simultaneously perform LVO detection and affected vessel classification. Initial training demonstrated high sensitivity and low false positive rates for both tasks on the internal and independent test sets. We also trained the model using images cropped around the Circle of Willis (CoW), as LVOs in our dataset are typically located within these vessels or their immediate neighbors. Figures 4 and 6 show that using only the CoW regions as input achieves sensitivity comparable to using the full images.

The independent test set, CODEC-IV, differs substantially from our development dataset. It consists of CTP-derived CTA images, acquired on different scanners, with slice thickness ranging from *0.5 - 1* mm (compared to a maximum of 0.5 mm in our internal dataset). Moreover, bounding box annotations in CODEC-IV are uniform in size, precisely localizing the occlusion, whereas our internal dataset boxes vary in size and often capture the full extent of the LVO. Despite these differences, both global and local models achieved satisfactory detection performance on CODEC-IV using a 2% IoU threshold to account for the discrepancy in annotation sizes. Under this criterion, the global approach achieved 0.90 sensitivity at 0.50 false positives per image, and the local approach 0.88 sensitivity at 0.56 false positives per image.

For the internal test set, we investigated whether removing false positives located outside the ROI could improve performance. At a sensitivity of 0.97, this reduced the false positives per image from 0.20 to 0.17, but still did not outperform the local approach, which achieved 0.13 false positives per image at the same sensitivity. This suggests that most false positives occur within the Circle of Willis, and that the surrounding region could safely be excluded during training.

In terms of localization accuracy, the mean center-to-center distance between predicted and ground truth bounding boxes on the independent test set was 2.47 ± 1.13 mm for the global approach and 2.43 ± 1.23 mm for the local approach. On the internal test set, the corresponding distances were 1.76 ± 1.49 mm and 1.76 ± 1.32 mm for the global and local approaches, respectively. These results indicate that our models accurately predicted LVO locations even when bounding box sizes differed, and that the local approach maintains localization accuracy consistent with the global approach.

Direct comparisons with prior state-of-the-art methods are limited because our framework jointly performs detection and classification, whereas most existing methods address only one task. Nevertheless, compared to Brugnara et al. (2023), who trained nnDetection on 835 cases (including controls and a wide range of vessel occlusions) and achieved 0.94 sensitivity at 4 false positives per image, our models reached the same sensitivity at a lower false positive rate, despite using a smaller training set. Similarly, compared to Russo et al. (2026), who used a subset of data from our collaborating hospital and reported 0.708 sensitivity at 1 false positive per image, both our global and local models achieved higher sensitivity with substantially fewer false positives.

Classification performance, summarized in Figs. 5 and 7, was satisfactory. On the internal test set, accuracies of 94% and 91% were obtained for the global and local approaches, respectively, indicating minimal impact of cropping to the CoW ROI. On the independent test set, overall class accuracies were 87% and 81%, and Cohen’s kappa scores were 0.62 (global) and 0.40 (local), indicating substantial and fair-to-moderate agreement, respectively. For the internal dataset, almost perfect agreement was observed, with *κ =0.88* (global) and *κ =0.82* (local).

Our development dataset consisted only of LVO-positive cases. Therefore, to further investigate the model’s behavior on LVO-negative cases, we conducted a supplementary inference-only evaluation using the IACTA-EST challenge dataset, which provides binary LVO labels without bounding box annotations. This dataset was not incorporated into training due to differences in its preprocessing protocol relative to our development pipeline, and because it provides only a global binary label indicating the presence or absence of LVO, rather than the bounding box annotations required for detection training. Both the global and local ensemble models were applied to all 116 cases (65 LVO-positive, 51 LVO-negative), and the highest confidence score per case was recorded. The global model produced a mean confidence of 0.79 ± 0.20 for LVO-positive cases and 0.32 ± 0.13 for LVO-negative cases. The local model produced 0.57 ± 0.26 and 0.26 ± 0.13, respectively. Using the maximum confidence score per case to discriminate between LVO-positive and LVO-negative cases, we obtained an AUC of 0.95 for the global approach and 0.84 for the local approach. These results demonstrate a strong discriminative ability between positive and negative cases, suggesting that an appropriate confidence threshold could help limit false alerts in a clinical triage setting. We note, however, that this evaluation is limited by the absence of bounding box annotations in the IACTA-EST dataset and by differences in preprocessing, and that a prospective evaluation on a clinically representative cohort remains necessary.

Our study has some limitations. The primary limitation is that the training dataset was collected at a single center using the same scanner and consists of a relatively small number of cases, which may limit the model’s exposure to variability in image acquisition and patient populations. Nevertheless, the models performed satisfactorily on an independent dataset from a different source. For future work, we plan to expand the dataset to include multiple clinical sites and a wider variety of LVO locations, which could further enhance model performance and generalizability. Another limitation is the class imbalance present in both the training and testing cohorts, particularly for basilar artery occlusions, which are substantially less represented than anterior circulation occlusions. Although this distribution reflects the natural prevalence of LVO locations reported in clinical practice, the relatively small number of basilar artery cases in the internal test set limits the statistical robustness of class-specific classification results for this minority vessel category.

Finally, it is worth noting the computational efficiency of our approach. Although absolute execution times depend on the hardware and implementation, the local approach, which restricts input to the Circle of Willis region, is approximately 3.3 times faster than the global approach, with mean inference time of 89.17 ± 13.67 seconds per case for the local approach against 227.14 ± 25.17 seconds per case for the global approach. This reduction in processing time could be particularly advantageous in acute stroke settings, where faster inference may contribute to more rapid diagnosis and treatment planning.

In summary, both the global and local approaches demonstrated excellent performance in detecting LVOs and classifying the affected vessel segments. While the local approach offers comparable accuracy to the global approach, its primary advantage is the reduced computational cost. By focusing on the Circle of Willis region during inference, it provides a more efficient solution without compromising clinical performance, potentially improving workflow and patient outcomes in acute stroke care.

## Conclusion

In this work, we introduced an approach for simultaneous Large Vessel Occlusion (LVO) detection and vessel classification using the nnDetection framework. While previous studies have demonstrated the framework’s effectiveness for LVO detection, this is the first study to address both detection and vessel-level classification simultaneously. Our results show strong performance in both tasks by leveraging spatial information from vessels near the Circle of Willis, guiding the network to the most relevant regions of brain CTAs.

We compared a localized training approach focused on the Circle of Willis region with a global approach using full-sized images, finding that both yielded comparable results. Evaluation on an independent test set demonstrated that the models can accurately localize occlusions in unseen cases, as supported by physical center-to-center distance measurements, although different annotation protocols limit the direct comparability of IoU. On the in-house test set, the global approach achieved a sensitivity of 0.97 at 0.20 false positives per image, while the local approach reached the same sensitivity with only 0.13 false positives per image. For classification, accuracies were 94% for the global approach and 91% for the local approach. Additionally, the local approach reduced processing time per case by a factor of three.

In conclusion, both approaches delivered comparable performance, as confirmed by independent testing. Importantly, training and inference restricted to the region of interest significantly lower computational costs without sacrificing accuracy, a feature particularly relevant for clinical stroke workflows that demand rapid image analysis.

## Information Sharing Statement

The in-house dataset used for developing of the algorithm in the current study is not publicly available due to the confidentiality policy and institutional patient privacy regulation. The CODEC-IV dataset can be obtained in accordance with the original data provider’s and license terms (Werdiger et al., 2023). The method described in this paper is implemented based on publicly available deep learning framework nnDetection (https://github.com/MIC-DKFZ/nnDetection). The code for the Circle of Willis segmentation pipeline used in this project is publicly available at https://github.com/NIC-VICOROB/CoW-multiclass-segmentation-TopCoW24

## Data Availability

The in-house dataset used for developing of the algorithm in the current study is not publicly available due to the confidentiality policy and institutional patient privacy regulation. The CODEC-IV dataset can be obtained in accordance with the original data provider’s and license terms.

## Footnotes

1 https://lgiancauth.github.io/iacta-est-2023/
